# Sycotic Warts

**Published:** 1888-08

**Authors:** 


### SYCOTIC WARTS.

WARTS in general: Ambr., *Amm-c.*, Anac., Ant-cr., *Ars.*, *Bar.*, BELL., Berb., Borax, Bov., CALC., Carb-an., Carb-v., CAUST., Chel., Con., Cupr., DULC., *Euph.*, *Euphr.*, *Hepar*, *Kali-c.*, Lach., *Lyc.*, *Nat-c.*, *Nat-m.*, NIT-AC., Petr., *Phos.*, *Ph-ac.*, *Rhus*, *Ruta*, Sabin., Sars., *Sep.*, *Sil.*, Spig., *Staph.*, SULPH., Sul-ac., THUJA.

### CHARACTER.

— beating (pulsating): *Calc.*, Kali-c., Lyc., Nit-ac., Petr., Sep., *Sil.*, Sulph.
— bleeding: *Nat-c.*, Nit-ac., Thuja.
— burning: Ars., Lyc., *Petr.*, *Phos.*, *Rhus*, Sep., Sulph., Thuja.
— flat: *Dulc.*, Lach.
— — hard and brittle: Ant-cr.
— fleshy or seedy: Caust., Nat-m.
— hard: *Ant-cr.*, Borax, Dulc., Ran-b., Sil., *Sulph.*
— horny; *Ant-cr.*, Borax, Ran-b., *Sulph.*, *Thuja.*
— indented: Calc., Euphr., Lyc., Nit-ac., *Ph-ac.*, *Rhus*, Sabin., Staph., *Thuja.*
— inflamed: Amm-c., Bov., *Calc.*, *Caust.*, Hepar, Lyc., *Nat-c.*, *Nit-ac.*, Rhus, Sep., *Sil.*, Staph., *Sulph.*
— itching: Euphr., Kali-c., Nit-ac., Phos., Sulph., Thuja.
— large: *Caust.*, *Dulc.*, Nat-c., *Nit-ac.*, Kali-c., *Sep.*
— old: *Calc.*, *Caust.*, *Nit-ac.*, *Rhus*, *Sulph.*
— painful: *Caust.*, *Nat-c.*, Nat-m., Nit-ac., Ruta, Sabin., Sulph., Thuja.
— — as if sore: Hepar, Lact., Nat-m., Nit-ac., Sabin., Thuja.
— pedunculated: Dulc., *Lyc.*, THUJA.
— small: Bar., *Calc.*, Dulc., Hepar, Lach., *Rhus*, *Sars.*, Sep., Sulph., *Thuja.*

WARTS, Stinging: Ant-cr., Bar., *Calc.*, Caust., Euphr., Hepar, Lyc., *Nit-ac.*, Rhus, Sep., Sil., *Sulph.*, Thuja.
— suppurating, humid: Ars., Bov., *Calc.*, *Caust.*, Hepar, Sil., *Thuja.*
— tickling: Sulph., Thuja.
— ulcerated: *Ars.*, Calc., *Nat-c.*, Phos.

LOCATION.

Eyes, below: Sulph.
Eyebrows: *Caust.*
Eyelids: *Caust.*, Nit-ac.
Canthi: Nit-ac.
Nose: Caust.
Tongue, nodosities or warts on the: Mang-acet.
Face: *Caust.*, Dulc., Kali-c., Nit-ac., Sep.
Mouth: Calc-ph.
Arms: Ars., Calc., Caust., Dulc., Nat-c., Nit-ac., *Sep.*, *Sulph.*
— forearm: *Calc.*
Hands: Anac., Berb., Borax, *Calc.*, *Dulc.*, *Lach.*, *Lyc.*, *Nat-c.*, Nat-m., *Nit-ac.*, Phos., Rhus, *Sep.*, *Sulph.*, *Thuja.*
— palms: Anac., *Nat-m.*, Ruta.
— fingers: Berb., Lach., Lyc., Petr., *Rhus*, *Sulph.*, Thuja.
— — ends of fingers: Caust., Thuja.
— — back of fingers: Dulc.
— — knuckles: Pallad., Selen.
— — sides of fingers: Calc.
— — thumb: Lach.

