# Erratum

**Published:** 1888-09

**Authors:** 


Erratum.—In August issue, page 434, under Warts, painful as if sore, for *Lact.* read *Lach.*

